# Development of a highly sensitive chemiluminescent enzyme immunoassay for fragmented cytokeratin 18 using new antibodies

**DOI:** 10.1038/s41598-021-97439-5

**Published:** 2021-09-14

**Authors:** Minori Yamada, Akiko Eguchi, Koji Okuno, Koji Sakaguchi, Tetsuji Yamaguchi

**Affiliations:** 1 Bio-Reagent Material Development, Bio-Diagnostic Reagent Technology Center, Sysmex Corporation, 4-3-2 Takatsukadai, Nishi-Ku, Kobe, Hyogo 651-2271 Japan; 2 Department of Gastroenterology and Hepatology, School of Medicine, Mie University, 2-174 Edobashi, Tsu, Mie 514-8507 Japan; 3 JST, PRETO, 4-1-8 Honcho, Kawaguchi, Saitama 332-0012 Japan; 4 Scientific Affairs, Sysmex Corporation, 1-3-2 Murotani, Nishi-Ku, Kobe, Hyogo 651-2241 Japan; 5 Manufacturing Technology Development 2, Reagent Production, Sysmex Corporation, 4-3-2 Takatsukadai, Nishi-Ku, Kobe, Hyogo 651-2271 Japan

**Keywords:** Biochemistry, Biotechnology, Biomarkers

## Abstract

Fragmented cytokeratin 18 (fCK18) released from epithelial cells undergoing apoptosis is widely studied in various diseases. However, fCK18 measurement is not utilized in clinical practice due to imprecise disease-state cutoff values. Therefore, we set out to generate new monoclonal antibodies (mAbs) and a recombinant fCK18 (rfCK18) calibrator in an effort to develop a highly sensitive chemiluminescent enzyme immunoassay (CLEIA). New capture mAb (K18-624) had a high binding ability compared to the current commercial antibody. New detection mAb (K18-328) recognized 323S-340G of CK18. A rfCK18 was expressed in the soluble fraction of *E. coli* when the N-terminal region (260 amino acid residues) of CK18 was truncated. Analysis of performance and measurement of human fCK18 were evaluated using K18-624 and K18-328 in a highly sensitive CLEIA. The coefficients of variation (CV) for within-run and between-day repeatability were below 10% and the recoveries were in the range of 15%. The detection sensitivity was 0.056 ng/mL. Serum fCK18 levels were significantly increased in non-alcoholic steatohepatitis (NASH) patients when compared to healthy individuals. Our new fCK18 mAbs showed high affinity and sensitivity. CLEIA using our new antibodies will be useful in measuring fCK18 in human blood thereby generating accurate clinical diagnoses of human liver diseases.

## Introduction

Cytokeratin 18 (CK18) is a member of the family of type I keratins and is a component of the intermediate filament (IF) of epithelial cells and various tumor cells. In apoptotic cellular death CK18 is cleaved at Asp237 by caspase-6, one of a family of intracellular proteases, yielding a 26-kD N-terminal fragment and a 22-kD C-terminal fragment. The 22-kD C-terminal fragment is further cleaved at Asp396 by caspase-3 and -7 into a 19-kD fragment^1^. Soluble CK18 has been shown in vitro to be released to the extracellular environment by cells undergoing apoptosis and thus circulating in the blood^2^. The commercially available monoclonal antibody M30 specifically reacts to the CK18 fragment cleaved at Asp396^1^ and has been widely used in various diseases as a biomarker reflecting apoptotic load. Indeed, using an M30 antibody ELISA kit, alterations in levels of CK18 have been reported in breast cancer^3^, colorectal cancer^4^, ischemic stroke^5^, traumatic brain injury^6^, GVHD^7^ and particularly in various liver diseases including NASH and acute-on-chronic liver failure (ACLF)^8^.

Nonalcoholic fatty liver disease (NAFLD) is currently the most common chronic liver disease worldwide and affects both children and adults^9,10^. NAFLD is a spectrum of conditions ranging from non-alcoholic fatty liver (NAFL), NASH to cirrhosis and hepatocellular carcinoma (HCC). Many reports indicate that the measurement of fragmented CK18 was useful as a non-invasive biomarker monitoring NAFLD activity^11,12^. Although the CK18 M30-ELISA could distinguish between NASH, NAFL or healthy individuals, several studies have reported that a clear line of delineation could not be obtained between a NAFL patient and a healthy individual. Furthermore, the current CK18 M30-ELISA kit is not readily translatable in the clinical setting due to variations in disease marker cut-off values and diagnostic performance issues^13,14^. Therefore, a market exists for the development of a new highly sensitive assay to detect CK18 fragments with diminished background signal^15^.

In the present study, we generated new monoclonal antibodies, so called K18-624 and K18-328, which detect fCK18. In addition, we established a highly sensitive CLEIA for fCK18.

## Results

### Characterization of new antibodies

**Figure 1:**
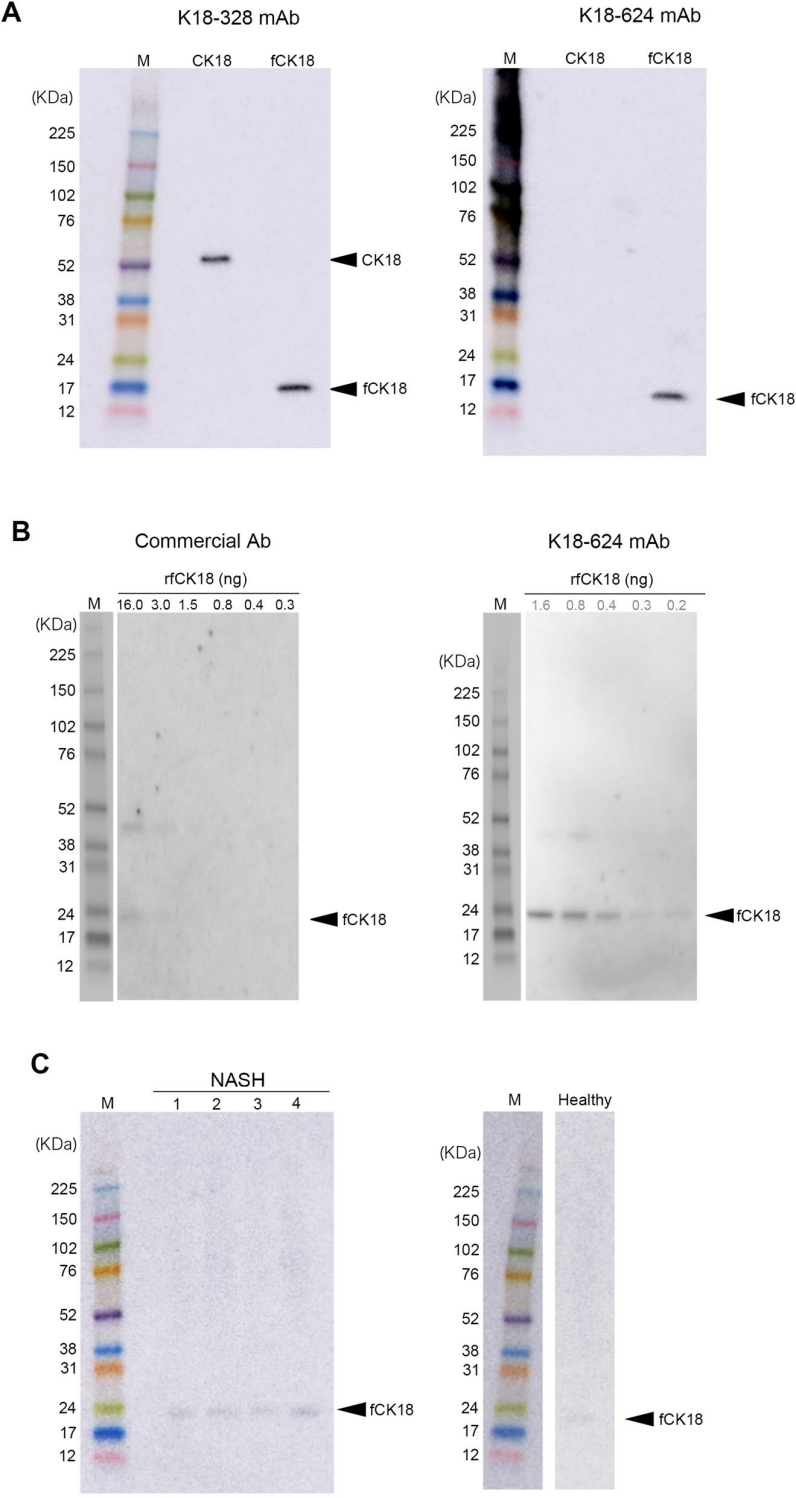
Characterization of antibodies by immunoblotting. (**A**) The detection of full-length CK18 (CK18) and fragmented CK18 (fCK18) with K18-328 antibody (left panel) or K18-624 antibody (right panel) by western blotting (WB). The arrows indicate the band of CK18 or fCK18. (**B**) The reactivity of antibodies, 22.5 ng of commercial (left panel) or 22.5 ng of K18-624 (right panel), to the different concentrations of rfCK18 by Immunoprecipitation-WB. The arrow indicates the band for rfCK18. A faint back between the 38 and 52 kDa marker indicates the band for dimer of rfCK18. (**C**) The detection of fCK18 using the K18-624 antibody and NASH patients or healthy individual by WB. The arrow indicates the band for fCK18.

To develop a highly sensitive CLEIA for fCK18, we generated new fCK18 monoclonal antibodies (mAb), called K18-624 and K18-328. K18-328 mAb identified both the CK18 protein and fCK18 (Fig. 1A, left panel), while K18-624 mAb identified fCK18 only (Fig. 1A, right panel) without reactivity to the full length CK18 protein (Fig. 1A, right panel). We further investigated overall reactivity of the K18-624 mAb to fCK18 using IP-WB and the recombinant fCK18 protein. The fCK18 bands were barely visible when using 16.0 ng of recombinant fCK18 (rfCK18) and a commercially available antibody (Fig. 1B, left panel, Supplementary Fig. S1A). In contrast, when using the K18-624 mAb fCK18 bands were faintly visible at 0.2 ng of rfCK18, which was one-eighth the concentration of the commercially available antibody yet the same level of detection, and markedly visible at 1.6 ng of rfCK18 (Fig. 1B, right panel, Supplementary Fig. S1A), indicating that the reactivity of the fCK18-624 antibody was approximately 8 times higher than the commercially available antibody. Furthermore, the K18-624 mAb identified fCK18 in serum from four NASH patients (Fig. 1C, left panel) and one healthy individual (Fig. 1C, right panel, Supplementary Fig. S1B). The commercially available antibody did not identify fCK18 (data not shown).

### Epitope analysis

**Figure 2:**
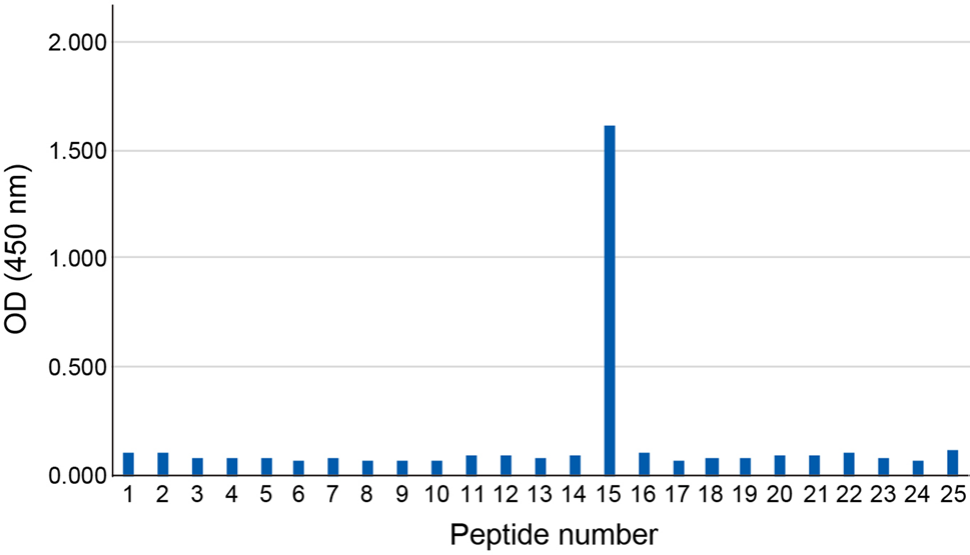
Epitope mapping of the K18-328 antibody. The reactivity of K18-328 antibody to a total of 25 peptides, which consist of a series of 158 overlapping peptides, each of which is 18 aa residues in length. Peptide number corresponds to the number in Table 1.

Based on the specificity of our new antibodies, we decided to use the K18-624 mAb as the capture antibody and the K18-328 mAb as a detection antibody for the highly sensitive CLEIA; therefore we deemed it necessary to perform epitope mapping for the K18-328 mAb. K18-328 mAb reacted with a series of 158 overlapping peptides, each 18 amino-acid residues in a length (Fig. 2, Table 1), and displayed a high reactivity to peptide 323–340 (Fig. 2). These results indicate that the K18-328 mAb recognizes an epitope at position 323–340 aa.

**Table 1:** Sequence of peptides for epitope mapping.

No	Peptide	Sequence of aa
1	239–256 aa	APKSQDLAKIMADIRAQY
2	245–262 aa	LAKIMADIRAQYDELARK
3	251–268 aa	DIRAQYDELARKNREELD
4	257–274 aa	DELARKNREELDKYWSQQ
5	263–280 aa	NREELDKYWSQQIEESTT
6	269–286 aa	KYWSQQIEESTTVVTTQS
7	275–292 aa	IEESTTVVTTQSAEVGAA
8	281–298 aa	VVTTQSAEVGAAETTLTE
9	287–304 aa	AEVGAAETTLTELRRTVQ
10	293–310 aa	ETTLTELRRTVQSLEIDL
11	299–316 aa	LRRTVQSLEIDLDSMRNL
12	305–322 aa	SLEIDLDSMRNLKASLEN
13	311–328 aa	DSMRNLKASLENSLREVE
14	317–334 aa	KASLENSLREVEARYALQ
15	323–340 aa	SLREVEARYALQMEQLNG
16	329–346 aa	ARYALQMEQLNGILLHLE
17	335–352 aa	MEQLNGILLHLESELAQT
18	341–358 aa	ILLHLESELAQTRAEGQR
19	347–364 aa	SELAQTRAEGQRQAQEYE
20	353–370 aa	RAEGQRQAQEYEALLNIK
21	359–376 aa	QAQEYEALLNIKVKLEAE
22	365–382 aa	ALLNIKVKLEAEIATYRR
23	371–388 aa	VKLEAEIATYRRLLEDGE
24	377–394 aa	IATYRRLLEDGEDFNLGD
25	383–397 aa	LLEDGEDFNLGDALD

*aa* amino acid residues.

### Recombinant fCK18 (rfCK18) proteins as a standard

**Figure 3:**
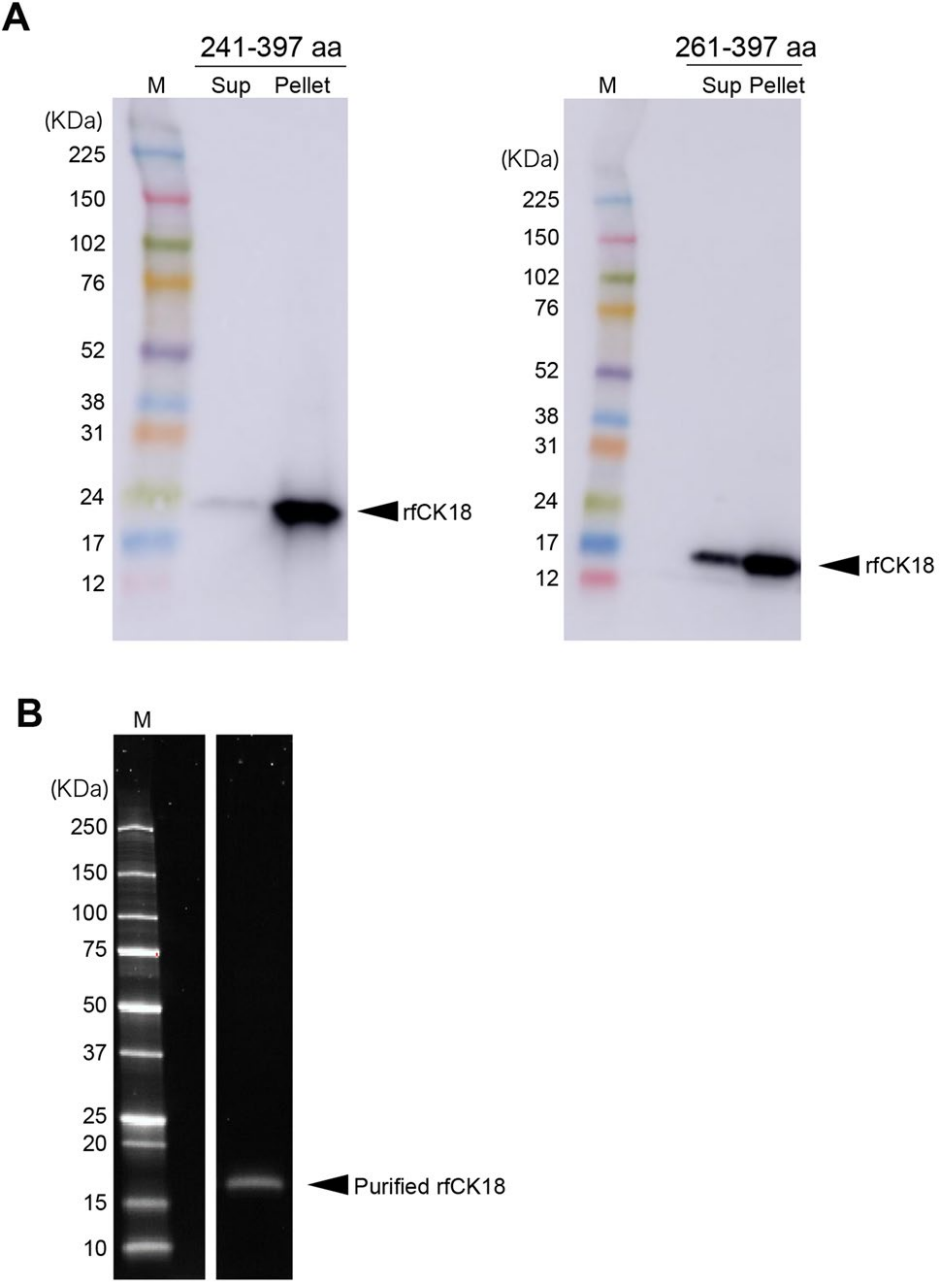
The expression of recombinant fCK18 in *E. coli.* (**A**) The immunoblot detection of recombinant fCK18 (rfCK18) proteins derived from 241 to 397 aa (left panel) and 261–397 aa (right panel) in *E. coli* culture supernatant (Sup) as a soluble protein and in the *E. coli* pellet (Pellet) as a inclusion body. The arrows indicate the band for rfCK18. (**B**) SDS-PAGE analysis of purified rfCK18 protein (261–397 aa) in the soluble fraction by fCK18–624 antibody conjugated affinity column chromatography. The arrow indicates the band for rfCK18.

rfCK18 protein (239–397 aa) was expressed as an inclusion body within *E. coli*, thus two other rfCK18 proteins were generated: 241–397 aa with deleted alanine and proline and 261–397 aa with deleted hydrophobic amino acid region. A portion of the rfCK18 protein (241–397 aa) was released in the soluble fraction (Fig. 3A, lane 1), but the majority of protein was observed within the insoluble fraction (Fig. 3A, lane 2). In contrast, the rfCK18 protein (261–397 aa) was released within the soluble fraction (Fig. 3A, lane 3), however some rfCK18 protein was still detectable within the insoluble fraction (Fig. 3A, lane 4). Based on these results, the soluble fraction of the rfCK18 protein (261–397 aa) was purified using fCK18-624 antibody conjugated affinity column chromatography (Fig. 3B, lane 1, Supplementary Fig. S2).

### Development of CLEIA for fCK18

**Figure 4:**
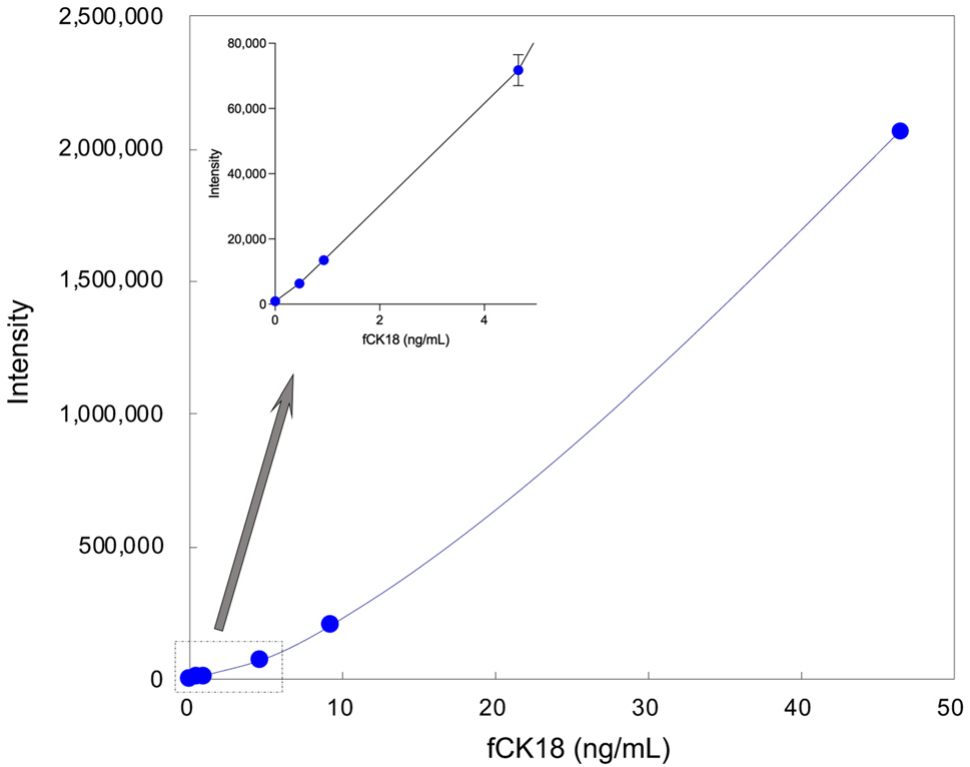
CLEIA for fCK18 measurement. The intensity range of fCK18 from 0.465 to 46.5 ng/mL based on our highly sensitive CLEIA.

We developed a highly sensitive CLEIA using HISCL-5000 CLEIA system that incorporates the K18-624 mAb conjugated magnetic beads as a capture antibody, ALP conjugated K18-328 Fab, which was produced by digestion of K18-328 mAb with pepsin following reduction, as a detection antibody, and rfCK18 protein as a standard. The standard range for the application was 0.465–46.5 ng/mL (Fig. 4) with 0.056 ng/mL being the limit of quantitation (LoQ) and 0.056 ng/mL being the limit of detection (LoD). Within-run reproducibility coefficient of variation (n = 10) was below 2% within the chosen range, with a low value of 1.1%, a middle value of 1.3%, and a high value of 1.2%. In addition, between-day reproducibility coefficient of variation (n = 5) was below 5% within the chosen range, with a low value of 1.7%, a middle value of 3.4%, and a high value of 4.2%. There was no detection of the full length CK18 protein in this system (data not shown).

### Validation of fCK18 measurement using human samples

**Figure 5:**
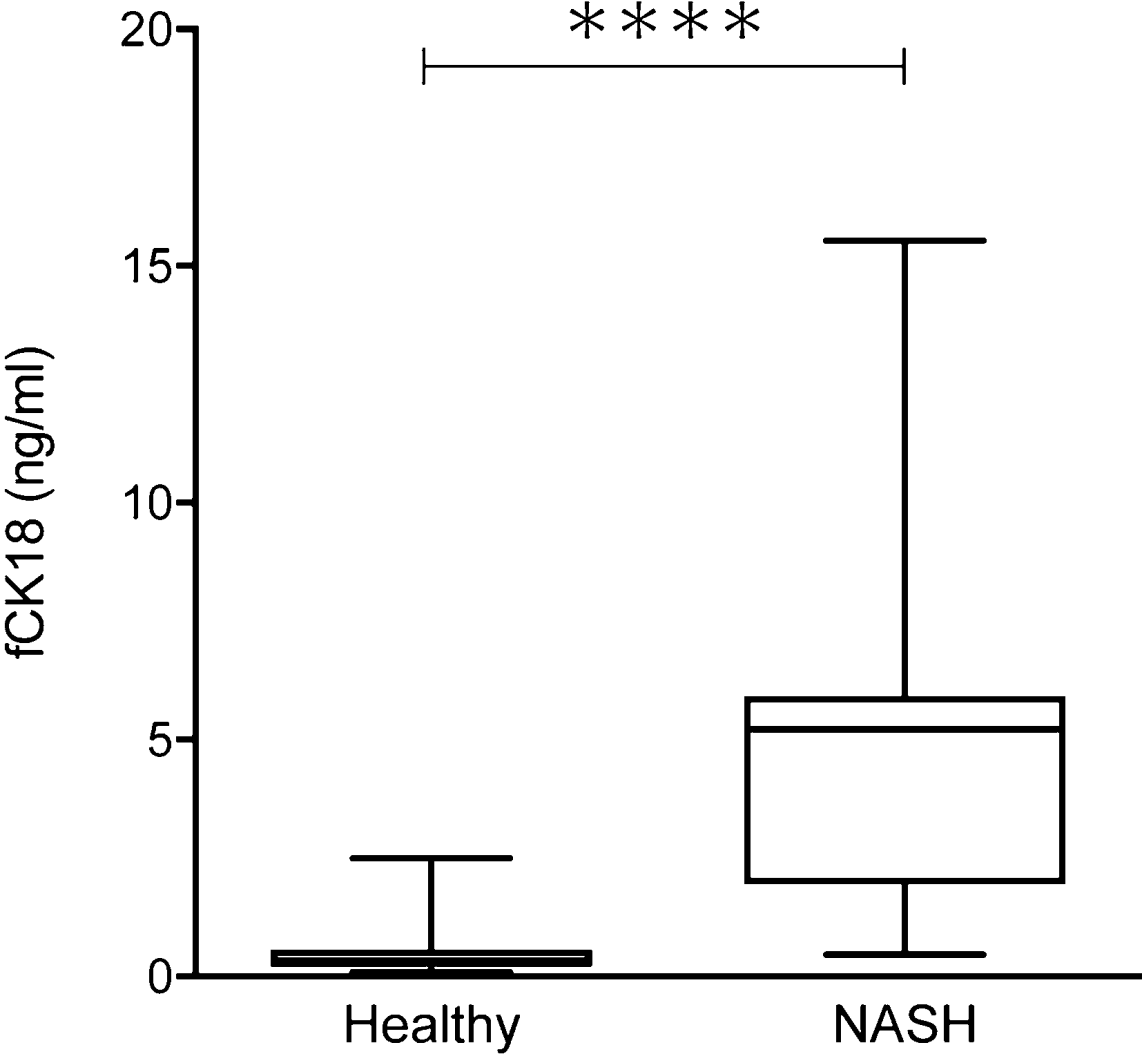
Distribution of serum fCK18 levels in healthy individuals and NASH patients. The measurement of serum fCK18 in 100 healthy individuals and 11 NASH patients using our highly sensitive CLEIA. *NASH* nonalcoholic steatohepatitis.

We concluded our measurements of fCK18 concentration in human samples using healthy individuals and NASH patients and the highly sensitive CLEIA. The levels of serum fCK18 were significantly increased in NASH patients when compared to healthy individuals (P < 0.0001) (Fig. 5). The range of observed serum fCK18 levels were between 0.087 and 15.531 ng/mL with 0.087–2.501 ng/mL being the range for healthy individuals, while NASH patients had a range of 0.460 ng/ml to 15.531 ng/mL, with a peak of 5.219 ng/mL (Fig. 5). This result indicates that the highly sensitive CLEIA was successful in measuring fCK18.

## Discussion

In the current study, we generated new fCK18 monoclonal antibodies with high affinity and sensitivity when compared to the currently available antibody. We also developed a highly sensitive CLEIA for measurement of fCK18 using these new antibodies.

Our new monoclonal antibody, K18-624, has a high reactivity and specificity as a capture antibody when compared to a commercially available antibody. The commercially available antibody is antigen generated using the supernatant from apoptosis-induced cells and recognizes an epitope in 387E-396D of CK18^16^. In contrast, K18-624 was antigen generated using a synthetic peptide, thus recognizing an epitope in 381R-397D, which we confirmed by western blotting (Fig. 1A). Furthermore, the 24 KDa band of fCK18 was detected in serum from healthy individuals and NASH patients by IP-WB using the fCK18-624 antibody (Fig. 1C, Supplementary Fig. S1B). By comparison, the detection of the 24 KDa band of fCK18 using the commercially available antibody was not observed (data not shown) and has yet to be reported. The differences in antigen source and epitope targeting leads to a high specify and reactivity for K18-624, which will recognize the three-dimensional structure of C-terminal fCK18, as compared to what we observed with the commercial antibody. When used as a detection antibody, our new K18-328 reacted with both fCK18 and full length CK18 and recognizes epitope 323S-340G of CK18 (Fig. 2). When we compare the commercially available detection antibody, called M5, it recognizes an epitope in 284T-396D based on the manufacturer’s instruction, or 300L-380T based on published data, suggesting that the targeted epitope may not be clearly identified. The combination of our new capture (K18-624) and detection (K18-328) antibodies enables the development of a highly sensitive CLEIA for fCK18.

fCK18 is generally expressed from region 239–397 aa of CK18^17^, thus we made a rfCK18 in E.coli transformed with an expression vector (239–397 aa). However, a rfCK18 protein derived from 239 to 397 aa was expressed as inclusion bodies in *E. coli*, therefore we explored other means by which to generate rfCK18 proteins. The majority of rfCK18 protein derived from 241 to 397 aa, deleting alanine and proline, was still expressed within inclusion bodies. In contrast, a rfCK18 protein derived from 261 to 397 aa, with a deleted hydrophobic amino acid region, was expressed as a soluble protein (Fig. 3A). To obtain a soluble rfCK18 protein derived from 239 to 397 aa we tried CHO cells, which are able to perform human-like posttranslational modifications and keep the tertiary structure of proteins. However, a rfCK18 protein (239–397 aa) from CHO cells aggregated on an SDS-PAGE gel (data not shown), which is unlike native fCK18 protein in mammalian blood that does not aggregate due to the presence of carrier proteins^18^. The strategy of making soluble rfCK18 protein devised in the current study will contribute to the production other soluble proteins which are mainly expressed as inclusion bodies.

In the measurement of fCK18 in human samples, the LoQ was 0.04 U/mL using the commercial kit according to manufacture's instruction, while it was 0.056 ng/mL (nearly equal to 0.006 U/mL) in our highly sensitive CLEIA. This result indicates that the measurement sensitivity of fCK18 was about sevenfold higher in our current system compared to the commercially available kit. There are several reasons as to why we were able to develop a highly sensitive CLEIA for fCK18: (1) our new capture antibody, K18-624 mAb, has a high sensitivity (Fig. 1B, Supplementary Fig. S1A), and (2) the HISCL system also has a high sensitivity especially when combined with the highly sensitive chemiluminescent substrates and the highly effective washing and isolation method (Bind/freemethod)^19,20^. The within-run reproducibility coefficient of variation and the between-day reproducibility coefficient of variation in the current study were better than those of the existing commercially available assay.

In the current study using our highly sensitive CLEIA, the fCK18 values were in the detectable range in all human samples, including healthy individuals and NASH patients, without any sample dilution. In previous published reports based on using a commercially available ELISA, the median value of CK18 fragment was 0.090–0.145 U/mL in healthy individuals^11,21^. These results reveal that our new highly sensitive CLEIA system could detect a lower concentration of fCK18 compared to the currently available assay system. We also confirmed that the concentration of fCK18 was significantly elevated in NASH patients, which is similar to findings from previous reports^11,12^, although we require further measurement of fCK18 in NASH patients with detailed clinical history. The measurement of CK18 fragment is recognized as a NASH biomarker and has been applied to several clinical trials in an effort to judge the effectiveness of new NASH medications^22–24^, but the existing ELISA has not been applied clinically due to variability of disease-related cut off values^13,14^. Based on the current results, our new highly sensitive CLEIA system has the potential to provide stable measurements of fCK18 with lower cut off values for healthy individuals and NASH patients which suggests application to clinical trials and the clinical setting would be appropriate.

In conclusion, we successfully developed a highly sensitive CLEIA using K18-624 and K18-328 antibodies for fCK18 measurement and demonstrated that our new highly sensitive CLEIA system is a simple and reliable detection apparatus when compared to the currently existing fCK18 ELISA. We also highlighted the potential of our highly sensitive CLEIA system in the clinical diagnosis of NASH.

## Methods

### Production of monoclonal antibodies

Monoclonal antibodies were prepared in a manner similar to that reported previously^25^. Briefly, mice were immunized with either recombinant CK18 protein (Prospec, Israel) or the synthetic peptide (**381**RRLLEDGEDFNLGDALD**397**) (BEX,Japan) and splenocytes of hyper-immunized mice were fused with myeloma cells (X 63). Positive clones were selected using ELISA with recombinant CK18 protein or the peptide. Large amounts of mAbs were purified using the HiTrap MabSelect SuRe system (Cytiva, Japan) in binding buffer (1.5 M glycine, 3 M NaCl, 10 mM EDTA (pH8.9)) and elution buffer (100 mM sodium citrate (pH 3.0)).

All methods were carried out in accordance with relevant guidelines and regulations. All experimental protocols were approved by the Institutional Animal Care and Use Committee at Sysmex corporation. This study was carried out in compliance with the ARRIVE guidelines.

### Recombinant fCK18 protein construction, design and purification

Two fragmented recombinant CK18 proteins were prepared according to an established method^25^. pBLC-fCK18 (241R-397D) and pBLC-fCK18 (261R-397D) were constructed by synthetic DNA into a pBLC expression vector (inhouse) using restriction enzymes, *NdeI* and *EcoRI* (Takara, Japan). Note: we defined the initiator protein, Methionine, as number 1 (1 M). Recombinant fCK18 expression used JM109 Escherichia coli (Wako, Tokyo, Japan); cells were transformed with pBLC-fCK18 (241R-397D) or pBLC-fCK18 (261R-397D), cultured at 37 °C in Luria Bertani (LB) medium until optical density at 600 nm reached 0.6, then further cultured at 37 °C for 12 h after induction with 500 mM isopropyl-b-d-thiogalactoside (Wako, Japan). Cells were recovered by centrifugation for 10 min at 10,000×*g* and kept at − 20 °C until subsequent analysis. The cells were sonicated in a lysis buffer (5 mM Tris–HCl (pH 8.0), inhibitor cocktail, and 0.2 mg/mL lysozyme) and soluble proteins were isolated by centrifugation at 10,000×*g* for 15 min. fCK18 (241R-397D) or fCK18 (261R-397D) were purified by affinity binding to a K18-624 mAb conjugated HiTrap NHS-activated HP column in buffer (Dulbecco’s phosphate-buffered saline) and eluted in buffer (0.1 M Glycine–HCl (pH 7.5)).The expression of fCK18 (241R-397D) or fCK18 (261R-397D) was assessed using SDS-PAGE and immunoblot analysis, and then fCK18 (241R-397D) or fCK18 (261R-397D) was desalted using a PD-10 column (Cytiva).

### SDS-PAGE, western blotting (WB), and immunoprecipitation (IP)

For SDS-PAGE, 0.2–16 ng of recombinant fCK18 was applied to a 4–20% Ready GEL (Bio-Rad, Japan) and stained by an immunofluorescence reagent (Oriole, Bio-Rad, Japan). Immunoblot analysis was carried out using 20 ng of recombinant CK18 and fCK18, applied on 4–20% Ready GEL (Bio-Rad), and transferred from the gel to a PVDF membrane. PVDF membrane was blocked with PVDF Blocking Reagent (TOYOBO, Japan) in Tris-buffered saline containing 0.05% Tween-20 (TBS-T) for 1 h at room temperature and incubated with K18-328 or K18-624 mAbs followed by a secondary HRP-conjugated antibody (MBL, Japan). Immunoreactive bands were visualized using HRP substrate reagents (Nacalai, Japan). For immunoprecipitation we covalently coupled K18-624 mAb or a commercially available antibody to magnetic beads (Magnosphere MS300/Carboxyl, JSR, Japan) using carboxyl and ethylene dichloride. The commercially available M30 antibody (VLVbio, Sweden) was diluted in 0.1 M MES buffer (pH 5.0) with beads and incubated for 1 h at room temperature; the beads were subsequently washed and stored in blocking buffer at 4 °C. Immune complexes were loaded equally on a 4–20% Ready GEL (Bio-Rad) and transferred from the gel to a PVDF membrane. PVDF membrane was blocked using a PVDF Blocking Reagent (TOYOBO, Japan) in Tris-buffered saline containing 0.05% Tween-20 (TBS-T) for 1 h at room temperature and incubated with alkaline phosphatase-conjugated K18-328 Fab. Immunoreactive bands were visualized using colorimetric alkaline phosphatase substrate reagents (Roche, Japan).

### CLEIA system

fCK18 in human serum was measured using the HISCL-5000 CLEIA system (Sysmex Corporation, Japan) that was developed using a two-step immunoassay system method and verified for human sera. Using this procedure, CK18-624 mAb conjugated magnetic beads were mixed with human serum, washed, and then allowed to react with alkaline phosphatase-conjugated K18-328 Fab. After the wash step, a chemiluminescent substrate (CDP-Star) was added and luminescence was emitted upon binding to the ALP. The amount of chemiluminescence was measured and its concentration was determined using recombinant fCK18 as a standard. The three different values, low, middle and high, were selected for CV. Low, middle, and high values were selected in the range of 0.465–0.930 ng/mL, 0.930–4.650 ng/mL, and 4.650–9.300 ng/mL from the fCK18 standard, respectively. LoD was defined as the point does not match between blank mean + 3 standard deviation (SD) and low concentration samples (such as a dilution of the lowest Std.) of mean − 3SD. LoQ was defined below 5% of the CV^26^.

All methods were carried out in accordance with relevant guidelines and regulations.

### Human samples

Serum from healthy individuals and NASH patients was purchased from Discovery Life Sciences (CA, USA) with approval and informed consent. Serum was stored at − 80 °C. fCK18 concentration was measured in 100 healthy individuals and 11 NASH patients.

All methods were carried out in accordance with relevant guidelines and regulations. All experimental protocols were approved by the Ethics Committee at Sysmex corporation.

### Statistical analysis

Continuous variables are presented as mean ± SD, and categorical variables are shown as number of patients. Data were analyzed using Manny–Whitney U test in two groups. All statistical analyses were performed using Prism (GraphPad Software, Inc. La Jolla, CA). Differences were considered to be significant at *P* < 0.05.

## Supplementary Information

Supplementary Information.

## Abbreviations

CLEIA: Chemiluminescent enzyme immunoassay
mAb: Monoclonal antibody
NAFL: Nonalcoholic fatty liver
NASH: Nonalcoholic steatohepatitis
