# Supplementary material for: Development of a highly sensitive chemiluminescent enzyme immunoassay for fragmented cytokeratin 18 using new antibodies
Source: Sci Rep. 2021 Sep 14;11:18187. doi: 10.1038/s41598-021-97439-5 (PMC8440549; doi:10.1038/s41598-021-97439-5)
Supplement: Supplementary file 1 — Supplementary Information. [file 41598_2021_97439_MOESM1_ESM.pptx]

## Slide 1
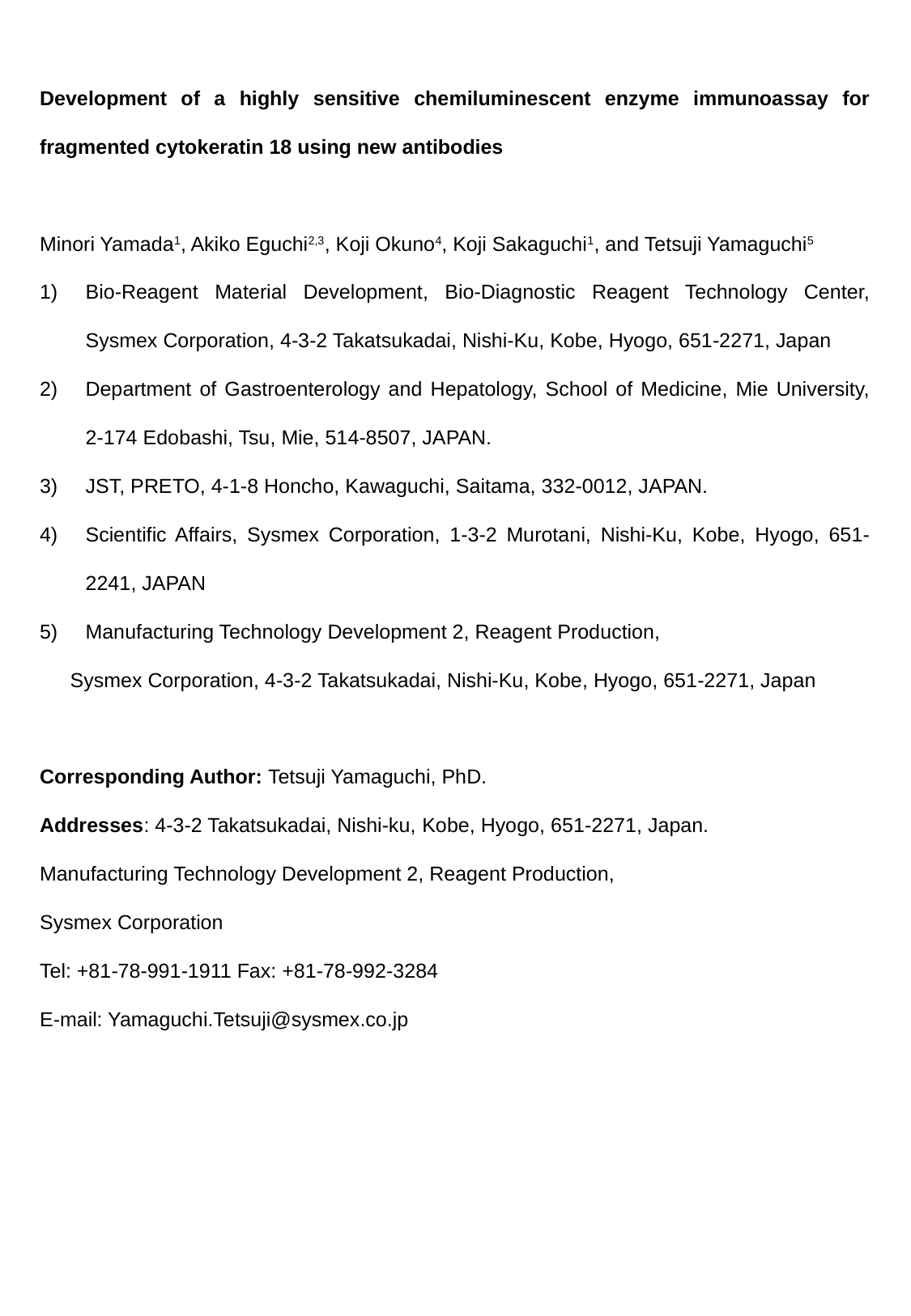

Development of a highly sensitive chemiluminescent enzyme immunoassay for fragmented cytokeratin 18 using new antibodies
Minori Yamada1, Akiko Eguchi2,3, Koji Okuno4, Koji Sakaguchi1, and Tetsuji Yamaguchi5
Bio-Reagent Material Development, Bio-Diagnostic Reagent Technology Center, Sysmex Corporation, 4-3-2 Takatsukadai, Nishi-Ku, Kobe, Hyogo, 651-2271, Japan
Department of Gastroenterology and Hepatology, School of Medicine, Mie University, 2-174 Edobashi, Tsu, Mie, 514-8507, JAPAN.
JST, PRETO, 4-1-8 Honcho, Kawaguchi, Saitama, 332-0012, JAPAN.
Scientific Affairs, Sysmex Corporation, 1-3-2 Murotani, Nishi-Ku, Kobe, Hyogo, 651-2241, JAPAN
Manufacturing Technology Development 2, Reagent Production,
Sysmex Corporation, 4-3-2 Takatsukadai, Nishi-Ku, Kobe, Hyogo, 651-2271, Japan
Corresponding Author: Tetsuji Yamaguchi, PhD.
Addresses: 4-3-2 Takatsukadai, Nishi-ku, Kobe, Hyogo, 651-2271, Japan.
Manufacturing Technology Development 2, Reagent Production,
Sysmex Corporation
Tel: +81-78-991-1911 Fax: +81-78-992-3284
E-mail: Yamaguchi.Tetsuji@sysmex.co.jp

## Slide 2
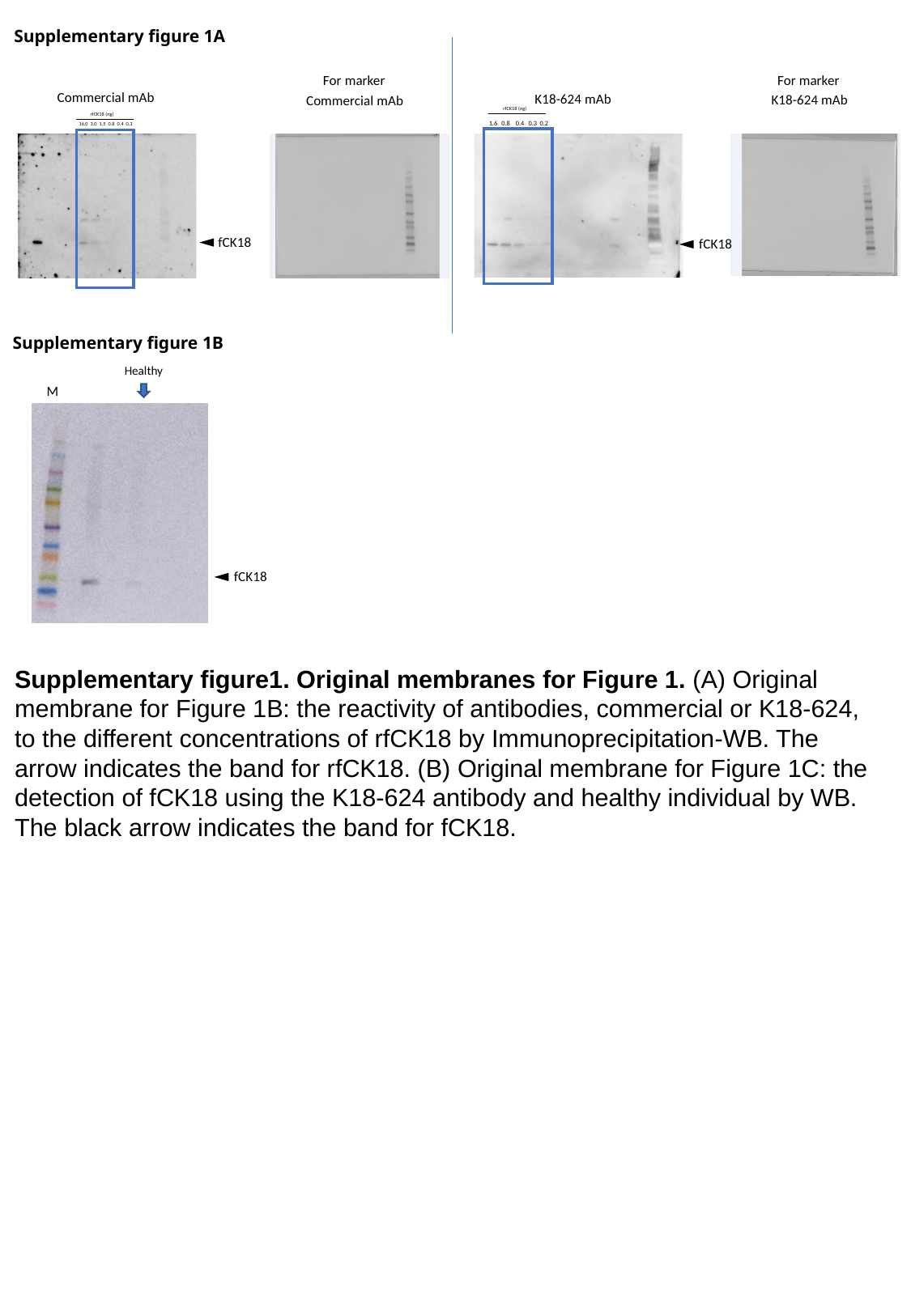

Supplementary figure 1A
For marker
For marker
Commercial mAb
K18-624 mAb
K18-624 mAb
Commercial mAb
rfCK18 (ng)
rfCK18 (ng)
1.6 0.8 0.4 0.3 0.2
16.0 3.0 1.5 0.8 0.4 0.3
fCK18
fCK18
Supplementary figure 1B
Healthy
M
fCK18
Supplementary figure1. Original membranes for Figure 1. (A) Original membrane for Figure 1B: the reactivity of antibodies, commercial or K18-624, to the different concentrations of rfCK18 by Immunoprecipitation-WB. The arrow indicates the band for rfCK18. (B) Original membrane for Figure 1C: the detection of fCK18 using the K18-624 antibody and healthy individual by WB. The black arrow indicates the band for fCK18.

## Slide 3
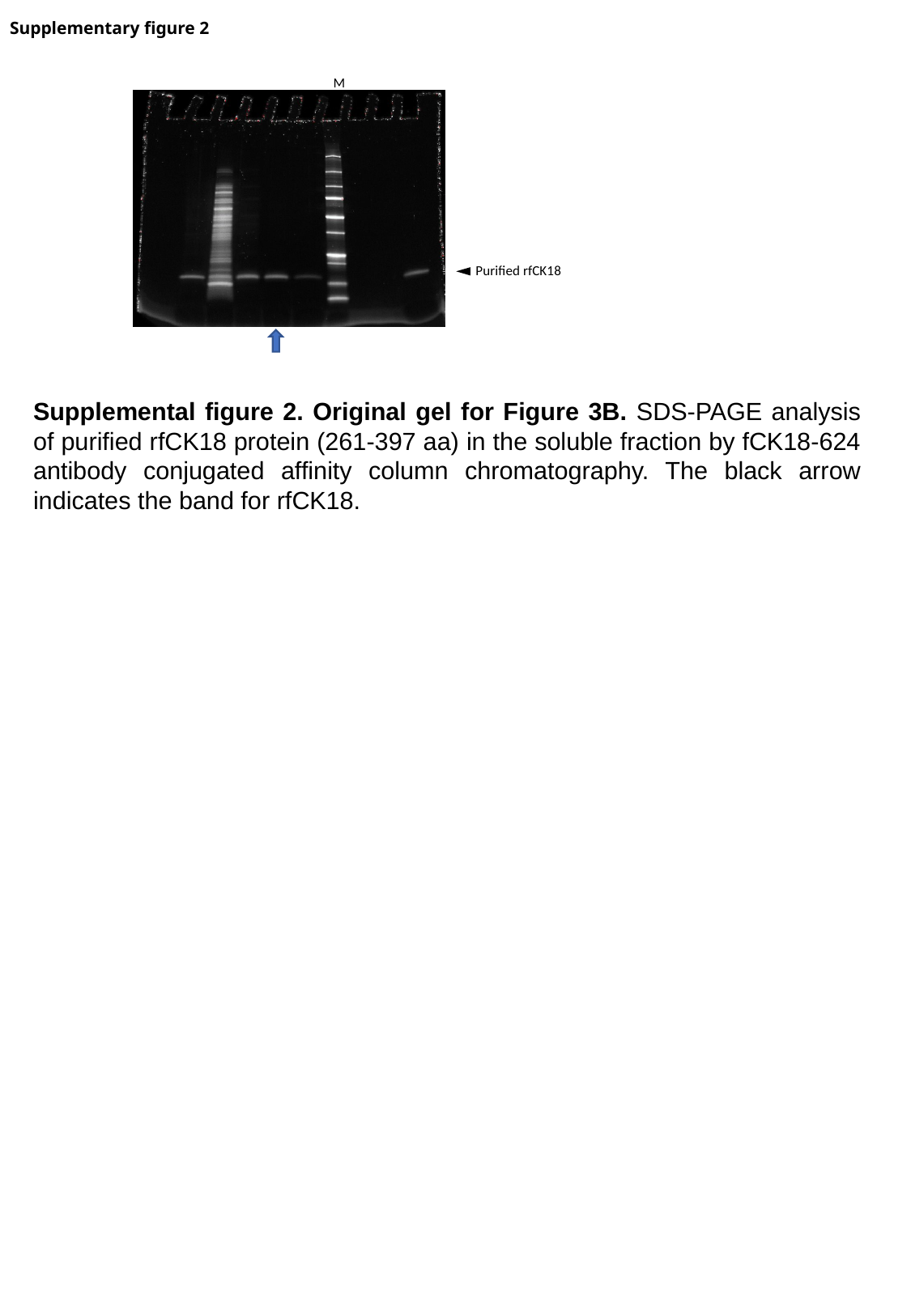

Supplementary figure 2
M
Purified rfCK18
Supplemental figure 2. Original gel for Figure 3B. SDS-PAGE analysis of purified rfCK18 protein (261-397 aa) in the soluble fraction by fCK18-624 antibody conjugated affinity column chromatography. The black arrow indicates the band for rfCK18.
